# Forensic DNA Barcoding and Bio-Response Studies of Animal Horn Products Used in Traditional Medicine

**DOI:** 10.1371/journal.pone.0055854

**Published:** 2013-02-08

**Authors:** Dan Yan, Jiao Y. Luo, Yu M. Han, Cheng Peng, Xiao P. Dong, Shi L. Chen, Li G. Sun, Xiao H. Xiao

**Affiliations:** 1 China Military Institute of Chinese Materia Medica, Military 302 Hospital, Beijing, China; 2 Institute of Medicinal Plant Development, Chinese Academy of Medical Sciences, Beijing, China; 3 College of Pharmacy, Chengdu University of Chinese Traditional Medicine, Chengdu, China; 4 Beijing Physical Examination Center, Beijing, China; Natural History Museum of Denmark, University of Copenhagen, Denmark

## Abstract

**Background:**

Animal horns (AHs) have been applied to traditional medicine for more than thousands of years, of which clinical effects have been confirmed by the history. But now parts of AHs have been listed in the items of wildlife conservation, which limits the use for traditional medicine. The contradiction between the development of traditional medicine and the protection of wild resources has already become the common concern of zoophilists, traditional medical professionals, economists, sociologists. We believe that to strengthen the identification for threatened animals, to prevent the circulation of them, and to seek fertile animals of corresponding bioactivities as substitutes are effective strategies to solve this problem.

**Methodology/Principal Findings:**

A powerful technique of DNA barcoding based on the mitochondrial gene cytochrome c oxidase I (COI) was used to identify threatened animals of Bovidae and Cervidae, as well as their illegal adulterants (including 10 species and 47 specimens). Meanwhile, the microcalorimetric technique was used to characterize the differences of bio-responses when those animal specimens acted on model organism (*Escherichia coli*). We found that the COI gene could be used as a universal primer to identify threatened animals and illegal adulterants mentioned above. By analyzing 223 mitochondrial COI sequences, a 100% identification success rate was achieved. We further found that the horns of Mongolian Gazelle and Red Deer could be exploited as a substitute for some functions of endangered Saiga Antelope and Sika Deer in traditional medicine, respectively.

**Conclusion/Significance:**

Although it needs a more comprehensive evaluation of bioequivalence in order to completely solve the problem of substitutes for threatened animals, we believe that the identification (DNA barcoding) of threatened animals combined with seeking substitutions (bio-response) can yet be regarded as a valid strategy for establishing a balance between the protection of threatened animals and the development of traditional medicine.

## Introduction

Threatened animal resources have been applied to traditional medicine for more than thousands of years [1], [2], [3], [4], [5], [6], [7]. Not only have their clinical effects got the validation of history, but also promote the development of traditional medicine. At the same time, the overuse of animal resources has accelerated the decline of threatened animals and even the extinction of some species (e.g., rhino) [8]. The international animal protection organizations such as the World Society for the Protection of Animals and the Wildlife Conservation Society, have attached great importance to the conservation of threatened animal resources, developing the Convention on International Trade in Endangered Species (CITES) and the establishment of the International Union for Conservation of Nature (IUCN) Red List. Those organizations hold that it is a destruction of biodiversity to inhumanly hurt and kill those threatened animals, which may even mean self-destruction in the long run [9].

As far as we know, animal-derived materials such as drugs, foods and raw stuffs are mostly existed as powders or compound preparations in the intermediate links such as trading markets and customs [10], [11]. It is often hard to identify those animal resources by routine methods such as the observation of appearance characters and the chromatographic analysis [12]. DNA barcoding, with its unique reproducibility, sequence versatility, and comparability among different species, provides a powerful approach to the authentication of species. Since the clinical efficacies of animal resources applied in traditional medicine have been confirmed by large numbers of patients, to prohibit the use of all animal resources at a time is impossible and unrealistic. Therefore, it is accessible and practical to find close phylogenetic species possessing corresponding bioactivities as substitutes of threatened animal resources, which is also commonly concerned by zoophilists, traditional medical professionals, economists, sociologists and so on. These efforts will greatly alleviate the burden on threatened species.

**Figure 1:**
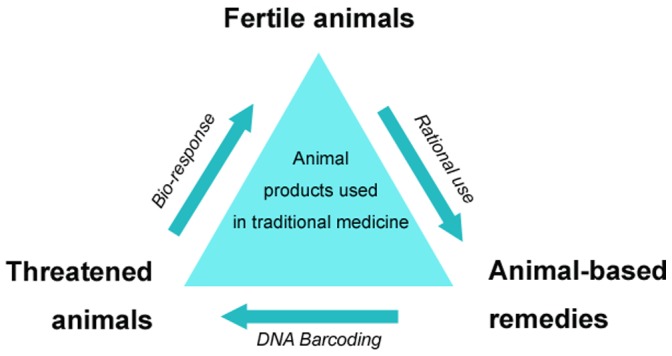
The strategy for threatened animals *VS* fertile animals used in traditional medicine.

Confronting the contradiction between the protection of threatened animals and the development of traditional medicine, we believe that to strengthen the identification for threatened animals, to prevent the circulation of them, and to seek fertile animals of corresponding bioactivities as substitutes are effective strategies to solve this problem (Figure 1).

The raw materials used in this study are listed in the “Convention on International Trade in Endangered Species of Wild Fauna and Flora”, “Wild Animals of Priority Protection in China” and the “Endangered Animal Species Red Book”. Ten animal-derived products including 47 samples from different animal species were investigated in this study. We investigated the use of DNA barcoding, a powerful molecular technique [13], [14], [15], [16], [17], [18], to discriminate between threatened and domestic animal species. We then conducted thermal active fingerprinting to evaluate the bio-response similarity of different animal horns (AHs) displayed bioactivity against model organisms (*Escherichia coli*). Use of DNA barcoding and thermal active fingerprinting, enabled us to evaluate the authenticity and bioactivity of AH. Therefore, we suggest that the identification (DNA barcoding) of threatened animals combined with seeking substitutions (bio-response) is a valid strategy for establishing a balance between the protection of threatened animals and the development of traditional medicine. The experimental strategy adopted for this work is shown in Figure 1.

## Results

### DNA Barcoding

PCR amplification success and sequencing reliability are important indices for evaluating potential DNA barcodes. In this study, both PCR amplification and sequencing of the DNA extracted from animal horns had 100% success rates. The amplicons obtained from amplification of the COI genes from the different samples are shown in Figure S1. From the 47 sequences analyzed by DnaSP, 487 sites were obtained, which after excluding sequence gaps or missing data comprised 482 sites. Of these, 169 sites (34.7%) were polymorphic. The nucleotide diversity (Pi) for this data set was 15.8%. The average nucleotide composition of all of the sequences was calculated in MEGA: T = 32.3%, C = 24.6%, A = 27.7%, G = 15.4%, with an average AT-richness of 60.0%.

Intra- and Inter-specific Genetic Divergence. For successful DNA barcoding, sequence variation must be high enough between species so that they can be discriminated from one another, but low enough within species that a clear threshold between intra- and inter-specific genetic variations can be defined. In this study, the mean intra- and inter-specific genetic variation was 0.2% and 15.8%, respectively. All of the intra-specific variation between sequences was <1.4%, whilst all of the inter-specific variation was >2.0%. Hence, the results of this analysis indicate that it is possible to identify and discriminate between the species from which the animal horns were obtained (using current DNA barcoding standards, i.e., >2% sequence divergence).

**Figure 2:**
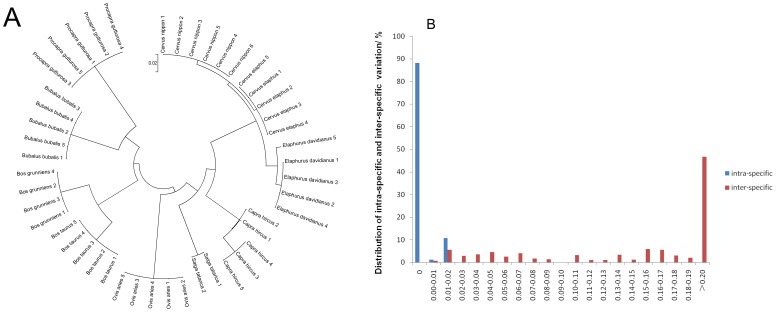
Analysis of the cytochrome c oxidase I (COI) DNA sequences. (**A**) Neighbor-joining tree with bootstrap analysis based on the kimura-2-parameter distances obtained for COI DNA sequences from 47 individual animal horns (Table 1); (**B**) Relative distribution of inter-specific and intra-specific variation.

Phylogenetic Analyses. The cladogram shown in Figure 2A was clustered based on the neighbor-joining (NJ) analysis of the animal horn samples obtained from the species shown in Table 1. The cladogram shows that all of the haplotypes grouped with their orthologous mitochondrial DNA sequences (Figure 2A). To test the accuracy of the barcodes and expand our identification capability, we downloaded close-related mitochondrial cytochrome c oxidase I (COI) genes available in GenBank. A phylogenetic analyses of 223 sequences was conducted, which included the GenBank dataset and our test sample dataset (Figure S2). The cluster branches indicated that all haplotypes strongly grouped with their orthologous mtDNA and every species clustered in the appropriate branch.

**Table 1:** The studied species, including scientific names, common names, family, sampling locations, and status in CITES legislation.

Scientific names	Common names	Family	Sampling locations	CITES legislation
*Cervus elaphus* * #	Red Deer	Cervidae	Jilin China	Appendix I
*Cervus nippon* * #	Sika Deer	Cervidae	Jilin China	NL
*Elaphurus davidianus* #	Père David's Deer	Cervidae	Beijing China	NL
*Saiga tatarica* * △	Saiga Antelope	Bovidae	Gansu China	Appendix II
*Procapra gutturosa* △	Mongolian Gazelle	Bovidae	Jilin China	NL
*Capra hircus* △	Domestic Goat	Bovidae	Sichuan China	NL
*Ovis aries*	Domestic Sheep	Bovidae	Sichuan China	NL
*Bos grunniens* ★	Domestic Yak	Bovidae	Tibet China	NL
*Bos taurus domesticus*	Domestic Cattle	Bovidae	Hena China	NL
*Bubalus bubalis* * ★	Asian Water Buffalo	Bovidae	Shandong China	NL

* : Referenced in Chinese Pharmacopeias or Japanese Pharmacopeias.

#, △, ★: The corresponding powder samples of animal horns are confusing in the market.

NL: not listed.

Barcoding Results. An ideal DNA barcode should have a conspicuous spacer region delineating intra- and inter-specific variation, which is called a “barcoding gap” [19], [20]. In this study, only a small proportion of the animal horn samples had overlapping intra- and inter-specific variation, hence it was possible to identify visible barcoding gaps (Figure 2B), indicating that genetic discrimination between the samples could be achieved with confidence.

The ability to unambiguously identify species in samples containing unrelated genetic material is one of the criteria for evaluating the quality of different barcodes. In this study, the taxonomic affiliation of each sequence (including the 47 test samples and the 176 individuals retrieved from GenBank) was determined by BLAST1 against the GenBank database. This analysis indicated that all of the mitochondrial sequences were successfully identified (Figure 2).

### Bio-response Evaluation

Biothermal profiles and similarities. The growth and metabolism of living organisms is accompanied by heat/energy production, which can be influenced by pathological changes or the action of drugs. Hence, it is possible to evaluate changes in microbial heat production in the presence or absence of different drugs using microcalorimetry. We assessed the biothermal profiles for the animal horn samples shown in Table 1.

The growth thermogenic curve for *E*. *coli* at 37 ^○^C in the absence of animal horn samples is shown in Figure S3. The HFP-*t* curve obtained here shows that the *E*. *coli* metabolic profile can be divided into two stages (stage 1 and stage 2) and five phases, i.e., a lag phase (A–B), the first exponential growth phase (B–C), a transition phase (C–D), the second exponential growth phase (D–E) and a decline phase (E–F).

**Figure 3:**
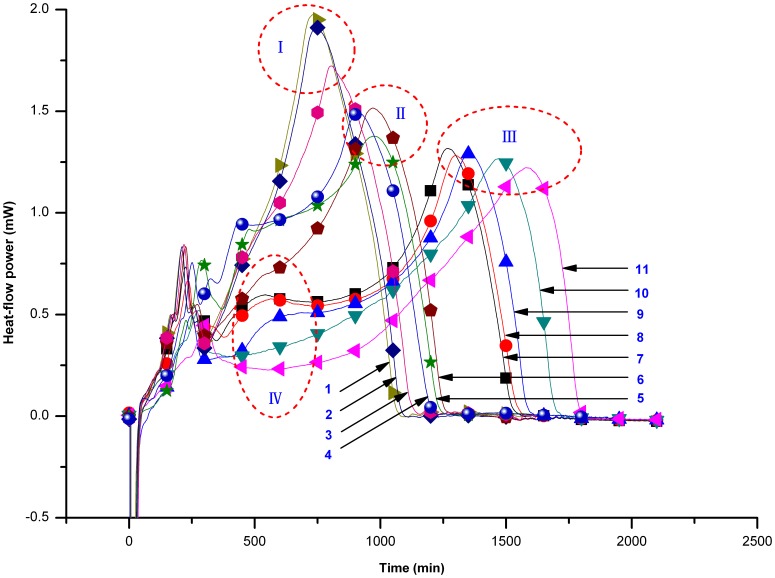
HFP-*t* curves in the presence of different animal horn products. The concentrations of the animal horn samples all were 25 mg/ml. 1- Red Deer. 2- Sika Deer. 3- Père David's Deer. 4- blank. 5- Asian Water Buffalo. 6- Domestic Cattle. 7- Domestic Yak. 8- Domestic Goat. 9- Domestic Sheep. 10- Mongolian Gazelle. 11- Saiga Antelope.

Similarly, the HFP-*t* curves for *E*. *coli* growth in the presence of different concentrations of the animal horn products from the different species tested were recorded and the curves corresponding to these experiments are shown in Figure 3. We can conclude from Figure 3 that the shape of the HFP-*t* curves changed when animal horn products were added to the *E*. *coli*. When using the same concentration of animal horn, the HFP-*t* curves profiles could be clustered to four distinct regions: region I, II, III and IV. Region I was primarily associated with *E*. *coli* heat output in the presence of the compounds derived from Cervidae animals (including Red Deer, Sika Deer and Père David's Deer). Region II and III are associated with inhibition of the heat output from *E*. *coli* in the presence of compounds derived from the Bovidae, within which group III (Caprinae and Antilopinae) exhibited stronger inhibition on *E*. *coli* growth than that of the group II animals (Bovinae). In addition, we found that Caprinae and Antilopinae, which were both classified as belonging to region III, shared a common characteristic in region IV, by which we could further distinguish the different compounds. For example, Mongolian Gazelle had no region IV profile, enabling it to be distinguished from Saiga Antelope.

**Figure 4:**
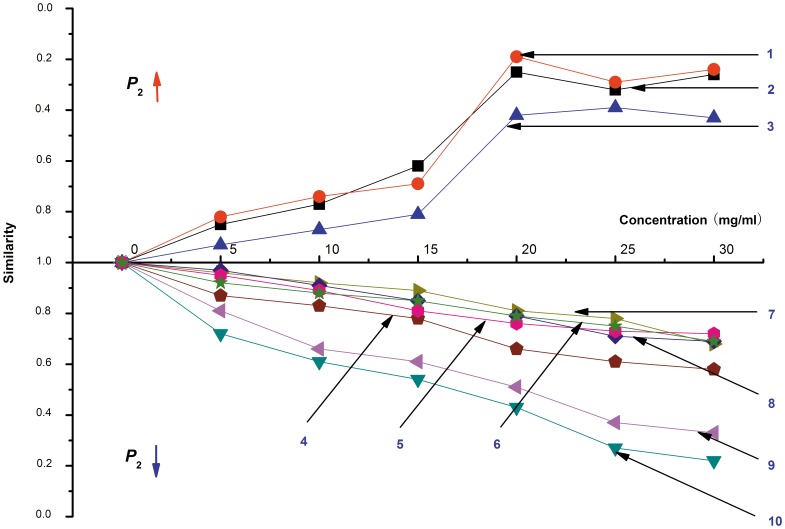
HFP-*t* curve similarities in the presence of different animal horn products compared with control. 1- Red Deer. 2- Sika Deer. 3- Père David's Deer. 4- Asian Water Buffalo. 5- Domestic Cattle. 6- Domestic Yak. 7- Domestic Goat. 8- Domestic Sheep. 9- Mongolian Gazelle. 10- Saiga Antelope.

To characterize the differences in the *E*. *coli* growth profiles in the presence of the different samples, the cosine method was applied to identify any similarities arising in the HFP-*t* curves. Figure 4 shows the similarities of the HFP-*t* curves for the 10 animal horn samples, compared with the *E*. *coli* growth curve without animal horn. From this analysis we noted that the compounds derived from Cervidae animals (Red Deer, Sika Deer and Père David's Deer) increased the total heat output from *E*. *coli* growth in comparison to the blank control. We also observed that Red Deer and Sika Deer had the most similar profiles, which suggests that Red Deer (currently listed in CITES legislation) might be substituted by Sika Deer (currently not listed in CITES legislation) for medical use. In contrast, the compounds derived from Bovidae animals including Caprinae (No. 7-9 curve on Figure 4), Antilopinae (No. 10 curve on Figure 4) and Bovinae (No. 4-6 curve on Figure 4) decreased the *E*. *coli* heat output in comparison to the blank control; the values of the similarity profiles were negative. Moreover, Caprinae and Antilopinae were similar to each other, but were dissimilar to Bovinae. It could be preliminary inferred from Figure 4 that Mongolian Gazelle could be exploited as a substitute for Saiga Antelope, however, Domestic Yak, Domestic Cattle and Asian Water Buffalo could not, despite being cheap and widely distributed in China.

Quantitative thermokinetic parameters for *E*. *coli* growth. The HFP-*t* curve for *E*. *coli* growth could be delineated by the following equation: *P_t_* = *P*_0_ exp(*kt*) or ln*P*_t_ = ln *P*_0_+ *kt*, where *P*_0_ and *P*_t_ represented the heat-flow power at time 0 or time (min), respectively. Using this equation, the growth rate constant *k*_1_ of the first exponential phase for *E*. *coli* growth at 37 ^○^C (in the absence of animal horn products) was calculated to be 0.0137±0.00031 min^−1^ (by analyzing the data for the first and second highest peaks). To test the reliability of the microcalorimetry, we repeated the experiment on eight occasions using the control bacteria and obtained good reproducibility. We next quantified thermokinetic parameters such as the heat-flow power of the first and the second highest peaks (*P*_1_ and *P*_2_), the appearance time of the first and second highest peaks (*t*_1_ and *t*_2_), the heat output for stage 1 and stage 2 (*Q*_sta,1_ and *Q*_sta,2_), and the total heat output (*Q*_t_) obtained from the HFP-*t* curves for *E*. *coli* growth in the presence of different concentrations of animal horn products (Table 2).

**Table 2:** Quantitative thermokinetic parameters for *Escherichia coli* growth at 37 ^○^C in the presence of different animal horn samples (25 mg/ml, n = 3).

Samples	*k*_1_(min^−1^)	*P*_1_(mW)	*T*_1_(min)	*Q*_sta,1_(J)	*k*_2_(min^−1^)	*P*_2_(mW)	*T*_2_(min)	*Q*_sta,2_(J)
blank	0.0137	0.762	246.8	4.56	0.00112	1.498	908.7	48.75
Red Deer	0.0152	0.825	212.1	5.95	0.00365	1.921	745.9	55.65
Sika Deer	0.0161	0.821	209.2	6.08	0.00376	1.975	732.1	58.02
Père David's Deer	0.0159	0.833	222.9	6.23	0.00275	1.723	803.1	53.87
Saiga Antelope	0.0082	0.443	304.5	7.04	0.00190	1.225	1566.2	41.35
Mongolian Gazelle	0.0092	0.514	278.6	6.78	0.00142	1.274	1504.3	44.25
Domestic Goat	0.0112	0.671	235.1	7.12	0.00189	1.285	1322.3	46.92
Domestic Sheep	0.0105	0.492	249.2	6.62	0.00122	1.286	1369.4	46.15
Domestic Yak	0.0119	0.729	223.5	6.87	0.00085	1.298	1290.5	47.95
Domestic Cattle	0.0161	0.846	225.7	6.18	0.00191	1.522	959.4	50.23
Asian Water Buffalo	0.0152	0.771	281.7	6.88	0.00095	1.374	972.5	49.84

The eight quantitative parameters (*k*_1_, *k*_2_, *P*_1_, *P*_2_, *t*_1_, *t*_2_, *Q*_sta,1_ and *Q*_sta,2_ in Table 2) were analyzed using PCA. The results indicated that *P*_2_ and *Q*_sta,2_ captured 92% of variation of Cervidae species, *k*_1_ and *t*_1_ captured 89% of variation of Bovidae species, therefore, their parameters were used for subsequent, respectively. A strong linear correlation between *k*_1_, *t*_1_, *P*_2_, *Q*_sta,2_ and the concentration (*c*) of the compound were obtained (Figure S4 and Figure S5).

## Discussion

It is our responsibility to protect threatened animals, however, the demand for traditional medicine is also an undeniable fact. We carry on this study through the identification for threatened animals combined with seeking for their substitutes. Taking some animals of Bovidae and Cervidae for examples, we preliminarily deem that the strategy based on DNA Barcoding and bio-response can establish a feasible balance between the protection of threatened animals and the development of traditional medicine. Because comparing with wild animal resources, the fertile animals with similar bioactivities can be propagated at large scales, and many of them are unused animal tissues from animals farmed for their meat (e.g., horn).

Molecular techniques have promoted the identification of animal-derived products; these techniques include protein electrophoresis, restriction fragment length polymorphism analysis, and using specific primers such as those for 12S rDNA, 16S rDNA, valine tRNA and Cyt B to amplify specific regions in the mitochondrial genome. Despite the great potential of genetics to assist in the identification of animal tissues and the phylogeny of animal clsdes [21], [22], [23], there has been little consensus on which gene region would be the most suitable. Recently, the mitochondrial cytochrome c oxidase I (COI) gene has attracted global attention as a DNA barcode for animals because the consortium for the barcode of life (CBOL) uses universal primers to amplify a region of the COI gene approximately 650 bp in length to identify broad animal taxonomy. This region is sequenced to give the DNA barcode for the specimen in question, and compared to barcodes from reference specimens to obtain a species identification [22], [24], [25]. Our results indicate that the COI gene is a useful barcode for the identification of animal products, in agreement with a previous report [14]; thus, this technique exhibits great potential for use in wildlife conservation.

In this study, we developed a DNA barcoding technique that can be used to discriminate between a wide range of animal species whose horns are used in traditional medicinal. We designed kits that enabled DNA to be extracted from cuticularized tissues, and adopted primers that universally amplified Bovidae and Cervidae family members. In addition, we improved the extraction process by adding silicon to remove proteins and organic compounds that may interfere with the DNA extraction and increasing the amount of samples used to increase the total amount of DNA extracted. The OD ratios (260/280) of all samples in our study were between 1.6 and 1.9. We determined that the COI gene could be used as a universal DNA barcode for discriminating between legitimate animal-derived compounds and counterfeit products. The development of additional DNA barcodes for identifying animal-derived compounds that share a common gene target would contribute significantly to the clinical application of animal-derived products.

Unlike living plants or fresh animal tissues, animal organs are often specially processed after their isolation from animals, e.g., by being airdried or dehydrated. In addition, the chemical components are quite complex, therefore traditional taxonomic tools and chromatographic methods may seem inefficient to identify. DNA barcoding has a low threshold and high efficiency, which makes it a sensible method for the identification of animal-derived compounds. The mean interspecies variances are 15–20 times larger than the mean intraspecies variances. Since high interspecies genetic variance is the premise of identifying species, we conclude that this fragment of the COI gene is efficient for the identification of the animal horns.

Threatened animals, chronically used in Asia, Africa [10], Latin America [26], South America [11], [27], [28], [29], [30], [31], and other places, play an important role in traditional medicine. It is unrealistic to prohibit the use of threatened animals if simply for protecting them. Therefore, a feasible solution for this problem is to find suitable substitutes from those close phylogenetic species of threatened animals. Then we used the technique of thermal active fingerprinting to characterize the differences of bio-responses of different animal specimens. The results showed that products derived from Cervidae animals increased the *E*. *coli* heat output; this finding is consistent with suggestions that animal horns from such animals have potential bioactivities connected with anti-aging and efficient gastrointestinal peristalsis. In contrast, products derived from Bovidae animals decreased the *E*. *coli* heat output, consistent with the belief that horns from such animals have potential antibacterial properties and can reduce fevers. The different AH samples had HFP-*t* curves specific for the animal family to which they belonged. We further found that the horns of Mongolian Gazelle and Red Deer could be exploited as a substitute for some functions of endangered Saiga Antelope and Sika Deer in traditional medicine, respectively. These substitutions could alleviate the burden on threatened species and increase the use of these substitutes in traditional medicines. In addition, since threatened animals may have various therapeutic benefits, their substitutes may only replace some certain functions but not the whole ones. Thereby, in order to confirm that whether a species could be used as a substitute for a threatened animal or not, it is necessary to comprehensively evaluate its main bioactivities even with works of clinical verification.

At present, there is little literature reported with respect to the situation of substitutes for threatened animals, such as pig bile instead of bear bile (Himalayan black bear, *Ursus thibetanus*, listed in IUCN Red List), jojoba oil instead of sperm oil (Sperm whale, *Physeter macrocephalus*, listed in IUCN Red List) and so on. Besides to find substitutes from close phylogenetic species of threatened animals, it is also considerable to find them from plant varieties [32], biological products [33], and even to prepare explicit active constituents of threatened animals using chemical synthesis. That may be an alternative to protect wild animal resources, although it often requires the support of massive and longstanding research efforts. We will pay sustained attention to this work. In particular, besides the use of threatened animals in traditional medicine, other factors such as the environmental change, the blind expansion of human activity, the decline of animal habitats, ornaments, totems, religious ceremonies and so on [34], [35], [36] are all result in animals in danger, all of which deserve our highly attention.

## Materials and Methods

### Sampling

A total of 47 individual specimens from 10 animal species were collected from seven Chinese states (Jilin, Beijing, Gansu, Sichuan, Tibet, Hena and Shandong). All the protected animal products were derived from the animals in National Nature Reserve; the fertile animals involved in this study were housed in the aquaculture bases. We have received a license from the State Forestry Administration of China to study the wildlife animals and the license number is “Lam Woo Approval [2005] 627”. The license number is available on the official website of the State Forestry Administration of China (http://www.forestry.gov.cn). The pilose antler (*Cervi Cornu Pantotrichum*) is sawed or cut off in spring or at the beginning of summer, boiled for a short while and airdried, and the other animal horns belonged to the renewable part of animals, which meant that these animals were not killed to obtain these samples.

The animal horns described here are derived from the wild animal species, including saiga antelope horn, *Cervus nippon* velvet antler and *Cervus nippon* antler were provided by the Animal Research Institute of the Chinese Academy of Sciences and China’s Forestry Sciences (Table 1).

### DNA Extraction, Amplification and Sequencing

DNA barcoding provides a powerful technique for identification of animal species, which helps in restricting the excessive use and illegal trade of such species. The technique mainly includes 3 portions: DNA extraction, amplification and sequencing.

Animal horns were shattered at −4°C and ground in a DNA extraction beveller (Retsch MM400, Germany) for 1 min at 1,800 rpm. The total DNA was extracted using E.Z.N.A. DNAout kits (Tiandz, Inc, Beijing, P.R. China), which extracted DNA from cuticularized tissue. PCR primers were designed using a ClustalW alignment of cytochrome c oxidase I (COI) GenBank sequences for the 47 species within Bovidae, Cervidae. The forward primer RonM_t1 TGTAAAAGGAGGGCCAGTGGMGCMCCMGATATRGCATTCCC and reverse primer VRl_t1 CAGGAAACAGCTATGACTAGACTTCTGGGTGGCCAAAGAAT CA, which was first suggested by Ivanova NV et al in 2007 [37], were adopted.

PCRs were performed in 25 µl volumes containing 13.3 µl of PCR-grade water, 2.5 µl of 10 × PCR buffer, 2.0 µl of magnesium chloride (2.5 mM), 1.0 µl of each primer (2.5 µM, Synthesized by Sangon Co., China), 2.0 µl of deoxynucleotide triphosphates (2.5 mM), 0.2 µl of Taq polymerase (5 units/µl, Biocolor BioScience & Technology Co., China), and 1.0 µl of template DNA (using approximately 30 ng of genomic DNA as a template in a 25 µl reaction mixture). The cycling conditions comprised an initial step of 3 min at 94°C, 33 cycles of 30 s at 94°C, 30 s at 58°C, and 60 s at 72°C, followed by 10 min at 72°C. The primers RonM_t1 and VRl_t1 were used to amplify a 487-bp fragment of the COI gene from each sample. The PCR products were electrophoresed in 1.0% tris-borate-EDTA agarose gels, stained with ethidium bromide, and visualized under ultraviolet light.

The PCR products were directly sequenced using the same primers in 20-µl reactions containing 2.0 µl of 10 × sequencing buffer (Applied Biosystems Co., USA), 1.0 µl of each primer (2.5 µM), 13.3 µl of PCR-grade water, 0.2 µl of BigDye (Applied Biosystems Co., USA), and 1.0 µl of PCR product. The cycling conditions comprised an initial step of 2 min at 95°C, followed by 30 cycles of 15 s at 96°C, 15 s at 52°C, and 4 min at 60°C. Sequencing reactions were performed in both directions on each DNA strand using the same PCR primers. Sequencing products were purified with Sephadex G-50 (Sigma-Aldrich Co., USA) columns in multiscreen HV filter plates (Millipore) and run on an Applied Biosystems ABI 3730XL DNA analyzer (Applied Biosystems Co., USA). The resulting sequences were assembled, edited, and aligned in Seq-Scape V.3.0 (Applied Biosystems, USA) before being uploaded to the Barcode of Life Data System.

### Bio-responses

Sample preparation for the bio-response studies. Animal horns are rich in keratin and polypeptides and are usually sold in powder form. The fine powder from the different AHs (2.0 g) was suspended in 15 ml of the artificial gastric juice (containing 0.03 M sodium chloride and 0.08 M hydrochloric acid). After that, the powder was ultrasonicated for 5 min and the suspension agitated by a bench top shaker (37°C) for 1 h. The supernatant obtained was used for analysis.

Living model. *Escherichia coli* (*E*. *coli*, CCTCC AB91112) was provided by the China Center for Type Culture Collection (Wuhan University, Wuhan, China). *E*. *coli* can be beneficial or harmful to animals. This bacterium coexists with other bacterial species in the intestinal tract. It has been suggested that the balance of gut microbiota is important for health and that traditional animal-derived drugs might have immunoloregulation, anti-fever, anti-bacterial and inflammatory responses on the intestinal tract. *E*. *coli* has been used as a model organism for studying the bioactivity of anti-fever and antibacterial drugs [38], [39], [40], [41]. Hence, we used *E*. *coli* as a high flux model for preliminary screening of the bioactivities of different animal-derived horns to establish their bio-response fingerprints. In the study, *E*. *coli* cultures were grown in 25 ml volumes in autoclaved medium containing peptone (10 g), beef extract (6 g) and sodium chloride (5 g) per 1000 ml (pH 7.0–7.2). The cultures were incubated aerobically with shaking at 120 rpm for 8 h at 37°C.

The bio-response experiments were performed using the ampoule method at 37°C with a 3114/3236 TAM air isothermal microcalorimeter (Thermometric AB, Sweden). *E*. *coli* were inoculated into 100 ml of Luria broth; the *E*. *coli* density was initially 1×10^6^ colony forming units (CFU) per ml. 10 ml of the bacterial suspension was added into each sterilized 20-ml glass ampoule. The animal horn solutions were diluted in 5 ml of methanol and ultrasonicated for 30 min. Different concentrations of the animal horn solutions were added to the bacterial suspension. Finally, each ampoule was sealed and placed into an eight-channel calorimeter block. When the temperature of ampoules reached 37°C, time-curves were recorded for each sample until the recorder returned to its baseline. All data were collected continuously using the dedicated software package (PicoLog TC-80, TA Corporation, USA) and “under peak” baseline reconstructions.

Microcalorimetry profile data are repeatable and provide real-time, online, dynamic information for characterization of the heat produced (small changes in thermal power) during vital life process in organisms, such as the exponential growth phase in *E*. *coli* and at cell death when all nutrients have been consumed [42]. We measured the following quantitative thermokinetic parameters: the growth rate constants (*k*_1_ and *k*_2_) of the first and second exponential phase, the heat-flow power of the first and the second highest peak (*P*_1_ and *P*_2_), the appearance time of the first and second highest peak (*t*_1_ and *t*_2_), the heat output in stage 1 and stage 2 (*Q*_sta,1_ and *Q*_sta,2_), and total heat output (*Q*_t_), from the heat flow power-time (HFP-*t*) curve. Curves were constructed for *E*. *coli* growth in the presence and absence of animal horn materials derived from Cervidae, Bovidae, Caprinae and Antilopinae.

### Data Analysis

DNA barcoding. The sequences were checked and merged using the CodonCode Aligner v 3.61 (CodonCode Co., USA) and aligned using ClustalX V 2.0 (Higgins D.G.). Wilcoxon signed rank tests were performed to compare the intra- and inter-specific variability for each pair of barcodes following the methods of Kress and Erickson [43]. The distribution frequency of inter- and intra-specific genetic divergences were calculated with the computer language Perl [44]. We evaluated DNA barcoding gaps by comparing the distribution of the intra-versus the inter-specific divergences [45]. Phylogenetic analyses were performed on 47 mitochondrial COI sequences. Haplotype distribution and munber of polymorphic sites were estimated with the software DnaSP 5.10.01 [46]. All taxa were subjected to pairwise sequence divergence calculations using the Kimura-2-parameter (K2P) model in MEGA 4 software [47], because this model can provide the best metric when genetic distances are low [48]. A neighbor-joining (NJ) tree with bootstrap analysis was constructed using MEGA 4. BLAST1, which was implemented using the BLAST program (Version 2.2.17), was used to search the reference database for each query sequence [49].

Bio-response fingerprinting. The cosine method [50], which uses the similarity calculating method of chromatography fingerprinting, was applied to evaluate the similarities between the *E*. *coli* HFP-*t* curves in the absence or presence of different concentrations of the processed animal horns.

Principal component analysis (PCA). As a standard data reduction technique, PCA extracts data, removes redundant information, highlights hidden features, and visualizes the main relationships that exist between observations among a large number of variables in terms of a smaller number of underlying factors (principal components or PCs) without losing very much information [51]. Here, PCA was performed on the parameters taken from the HFP-*t* curves to identify the main parameters.

## Supporting Information

Figure S1 **PCR amplication results of COI.** 01–05 Red Deer, 11–16 Sika Deer, 21–25 Père David's Deer, 31–32 Saiga Antelope, 41–45 Mongolian Gazelle, 51–54 Domestic Yak, 61–65 Domestic Cattle, 71–75 Asian Water Buffalo, 81–85 Domestic Goat, and 91–95 Domestic Sheep.
(TIF)

Figure S2 **Neighbor-joining tree of 223 COI complete gene sequences available at GenBank.** The blue circle represents the experimental individuals; the numbers in front of the taxon names are the species identification numbers.
(TIF)

Figure S3 **HFP-** ***t*** **curve in the absence of animal horn products.**
(TIF)

Figure S4 **Score plots generated from PCA of the eight quantitative parameters obtained from the HFP-** ***t*** **profiles. (A)** Score plots for Red Deer (main parameter *P*_2_ and *Q*_sta, 2_). **(B)** Score plots of Saiga Antelope (main parameter *k*_1_ and *T*_1_).
(TIF)

Figure S5 **Relationship between the main parameters and the concentration (** ***c*** **) of the animal horn samples.** **(A)** relationship between *P*_2_, *Q*_sta, 2_ and *c* for Red Deer. **(B)** relationship between *k*_1_, *T*_1_ and *c* for Saiga Antelope.
(TIF)

## References

[1] Alves RR, Rosa IL, Santana GG (2007) The role of animal-derived remedies as complementary medicine in Brazil. Bioscience 57: 949–955.

[2] Leal IR, Da Silva JMC, Tabarelli M, Lacher TE Jr (2005) Changing the course of biodiversity conservation in the caatinga of northeastern Brazil. Conservation Biology 19: 701–706.

[3] Mishra N, Rout SD, Panda T (2011) Ethno-zoological studies and medicinal values of similipal biosphere reserve, Orissa, India. Afr J Pharm Pharmaco 1: 6–11.

[4] Jacobo-Salcedo Mdel R, Alonso-Castro AJ, Zarate-Martinez A (2011) Folk medicinal use of fauna in Mapimi, Durango, México. J Ethnopharmacol 133: 902–906. 20937375 10.1016/j.jep.2010.10.005

[5] Alves RRN, Souto WMS, Barboza RRD (2011) Primates in traditional folk medicine: a world overview. Mammal Rev 40: 155–180.

[6] Alves RRN, Barboza RRD, Souto WMS (2010) A Global overview of canids used in traditional medicines. Biodivers Conserv 19: 1513–1522.

[7] Alves RRN, Vieira WLS, Santana GG (2008) Reptiles used in traditional folk medicine: conservation implications. Biodivers Conserv 17: 2037–2049.

[8] Haddad NM (2012) Connecting ecology and conservation through experiment. Nat Methods 9: 794–795. 22847112 10.1038/nmeth.2107

[9] Laurance WF, Useche DC, Rendeiro J, Kalka M, Bradshaw CJ, et al (2012) Averting biodiversity collapse in tropical forest protected areas. Nature 489: 290–294. 22832582 10.1038/nature11318

[10] Whiting MJ, Williams VL, Hibbitts TJ (2011) Animals traded for traditional medicine at the Faraday market in South Africa: species diversity and conservation implications. J Zool 284: 84–96.

[11] Oliveira ES, Torres DF, Brooks SE, Alves RRN (2010) The medicinal animal markets in the metropolitan region of Natal City, northeastern Brazil. J Ethnopharmacol 130: 54–60. 20460145 10.1016/j.jep.2010.04.010

[12] Coghlan ML, Haile J, Houston J, Murray DC, White NE, et al (2012) Deep sequencing of plant and animal DNA contained within traditional Chinese medicines reveals legality issues and health safety concerns. PLoS Genet 8: e1002657. 22511890 10.1371/journal.pgen.1002657 PMC3325194

[13] Schindel DE, Miller SE (2005) DNA barcoding a useful tool for taxonomists. Nature 435: 17. 10.1038/435017b 15874991

[14] Bitanyi S, Bjørnstad G, Ernest EM, Nesje M, Kusiluka LJ, et al (2011) Species identification of Tanzanian antelopes using DNA barcoding. Mol Ecol Resour 11: 442–449. 21481202 10.1111/j.1755-0998.2011.02980.x

[15] Hebert PD, Penton EH, Burns JM, Janzen DH, Hallwachs W (2004) Ten species in one: DNA barcoding reveals cryptic species in the neotropical skipper butterfly Astraptes fulgerator. Proc Natl Acad Sci U S A 101: 14812–14817. 15465915 10.1073/pnas.0406166101 PMC522015

[16] Magnacca KN, Brown MJ (2012) DNA barcoding a regional fauna: Irish solitary bees. Mol Ecol Resour 12: 990–998. 22931682 10.1111/1755-0998.12001

[17] Reid BN, LE M, McCord WP, Iverson JB, Georges A, et al (2011) Comparing and combining distance-based and character-based approaches for barcoding turtles. Mol Ecol Resour 11: 956–967. 21635698 10.1111/j.1755-0998.2011.03032.x

[18] Hebert PD, Cywinska A, Ball SL, deWaard JR (2003) Biological identifications through DNA barcodes. Proc Biol Sci 270: 313–21. 12614582 10.1098/rspb.2002.2218 PMC1691236

[19] Lahaye R, Bank M, Bogarin D, Warner J, Pupulin F, et al (2008) DNA barcoding the floras of biodiversity hotspots. Proc Natl Acad Sci USA 105: 2923–2928. 18258745 10.1073/pnas.0709936105 PMC2268561

[20] Meyer CP, Paulay G (2005) DNA barcoding: error rates based on comprehensive sampling. PLoS Biol 3: e422. 16336051 10.1371/journal.pbio.0030422 PMC1287506

[21] Guha S, Goyal SP, Kashyap VK (2007) Molecular phylogeny of musk deer: a genomic view with mitochondrial 16S rRNA and cytochrome b gene. Mol Phylogenet Evol 42: 585–597. 17158073 10.1016/j.ympev.2006.06.020

[22] Arif IA, Bakir MA, Khan HA (2012) Inferring the phylogeny of bovidae using mitochondrial DNA sequences: resolving power of individual genes relative to complete genomes. Evol Bioinform 8: 139–150. 10.4137/EBO.S8897 PMC3290115 22399841

[23] Hassanin A, Douzery EJ (2003) Molecular and morphological phylogenies of ruminantia and the alternative position of the moschidae.Syst Biol. 52: 206–228. 10.1080/10635150390192726 12746147

[24] Bitanyi S, Bjørnstad G, Ernest EM, Nesje M, Kusiluka LJ, et al (2011) Species identification of Tanzanian antelopes using DNA barcoding. Mol Ecol Resour 11: 442–449. 21481202 10.1111/j.1755-0998.2011.02980.x

[25] Reid BN, LE M, McCord WP, Iverson JB, Georges A, et al (2011) Comparing and combining distance-based and character-based approaches for barcoding turtles. Mol Ecol Resour 211: 956–967. 10.1111/j.1755-0998.2011.03032.x 21635698

[26] Alves RR, Alves HN (2011) The faunal drugstore: animal-based remedies used in traditional medicines in Latin America. J Ethnobiol Ethnomed 7: 1–43. 21385357 10.1186/1746-4269-7-9 PMC3060860

[27] Alves RRN, Rosa IL (2010) Trade of Animals Used in Brazilian Traditional Medicine: Trends and Implications for Conservation. Human Ecol 38: 691–704.

[28] Alves RR, Léo Neto NA, Brooks SE, Albuquerque UP (2009) Commercialization of animal-derived remedies as complementary medicine in the semi-arid region of Northeastern Brazil. J Ethnopharmacol 124: 600–608. 19422902 10.1016/j.jep.2009.04.049

[29] Alves RRN, Pereira Filho GA (2007) Commercialization and use of snakes in North and Northeastern Brazil: implications for conservation and management. Biodivers Conserv 16: 969–985.

[30] Alves RRN, Rosa IL (2006) From cnidarians to mammals: the use of animals as remedies in fishing communities in NE Brazil. J Ethnopharmacol 107: 259–276. 16621379 10.1016/j.jep.2006.03.007

[31] Alves RRN, Rosa IL (2007) Zootherapy goes to town: the use of animal-based remedies in urban areas of NE and N Brazil. J Ethnopharmacol 113: 541–555. 17719192 10.1016/j.jep.2007.07.015

[32] Feng YB, Wang N, Ng KM, Tsao SW, Nagamatsu T, et al (2009) Bear bile: dilemma of traditional medicinal use and animal protection. J Ethnobiol Ethnomed 5: 2. 19138420 10.1186/1746-4269-5-2 PMC2630947

[33] Ben-Nun IF, Montague SC, Houck ML, Tran HT, Garitaonandia I, et al (2011) Induced pluripotent stem cells from highly endangered species. Nat Methods 8: 829–831. 21892153 10.1038/nmeth.1706

[34] Cleland LG, James MJ, Proudman SM (2003) The role of fish oils in the treatment of rheumatoid arthritis. Drugs 63: 845–853. 12678571 10.2165/00003495-200363090-00001

[35] United States Food and Drug Administration, Public Health Service (1973) GRAS monograph series: Fish oils. Philadelphia: Tracor Jitco, Inc. 3–5.

[36] Benítez G (2011) Animals used for medicinal and magico-religious purposes in western Granada Province, Andalusia (Spain) J Ethnopharmacol. 137: 1113–1123. 10.1016/j.jep.2011.07.036 21801827

[37] Ivanova NV, Zemlak TS, Hanner RH, Hebert PDN (2007) Universal primer cocktails for fish DNA barcoding. Mol Ecol Notes 7: 544–548.

[38] Marschall J, Zhang L, Foxman B, Warren DK, Henderson JP (2012) Both host and pathogen factors predispose to *Escherichia coli* urinary-source bacteremia in hospitalized patients. Clin Infect Dis 16: 11–15. 10.1093/cid/cis252 PMC3357479 22431806

[39] Taglietti A, Diaz Fernandez YA, Amato E, Cucca L, Dacarro G, et al (2012) Antibacterial activity of glutathione-coated silver nanoparticles against gram positive and gram negative bacteria. Langmuir 16: 11–15. 10.1021/la3003838 22546237

[40] Jia W, Li HK, Zhao LP, Nicholson JK (2008) Gut microbiota: a potential new territory for drug targeting. Nat Rev Drug Discov 7: 123–129. 18239669 10.1038/nrd2505

[41] Lam W, Bussom S, Guan FL, Jiang ZL, Zhang W, et al (2010) The four-herb Chinese medicine PHY906 reduces chemotherapy-induced gastrointestinal toxicity. Sci Transl Med 45: 1–8. 10.1126/scitranslmed.3001270 20720216

[42] Wang F, Yao J, Chen H, Chen K, Trebse P, et al (2010) Comparative toxicity of chlorpyrifos and its oxon derivatives to soil microbial activity by combined methods. Chemosphere 78: 319–326. 19900695 10.1016/j.chemosphere.2009.10.030

[43] Kress WJ, Erickson DL (2007) A two-locus global DNA barcode for land plants: the coding *rbcL* gene complements the non-coding *trnH-psbA* spacer region. PLoS ONE 2: e508. 17551588 10.1371/journal.pone.0000508 PMC1876818

[44] Luo JY, Yan D, Zhang D, Han YM, Dong XP, et al (2011) Application of 12S rRNA gene for the identification of animal-derived drugs. J Pharm Pharmaceut Sci 14: 358–367. 10.18433/j3n017 21906480

[45] Kress WJ, Wurdack KJ, Zimmer EA, Weigt LA, Janzen DH (2005) Use of DNA barcodes to identify flowering plants. Proc Natl Acad Sci USA 102: 8369–8374. 15928076 10.1073/pnas.0503123102 PMC1142120

[46] Librado P, Rozas J, (2009) DnaSP v5: a software for comprehensive analysis of DNA polymorphism data. Bioinformatics 25, 1451–1452. 10.1093/bioinformatics/btp187 19346325

[47] Tamura K, Dudley J, Nei M, Kumar S (2007) MEGA4: molecular evolutionary genetics analysis (MEGA) software version 4.0. Mol Biol Evol 24: 1596–1599. 17488738 10.1093/molbev/msm092

[48] Nei M, Kumar S (2000) Molecular Evolution and Phylogenetics. Oxford University Press, Oxford.

[49] Ross HA, Murugan S, Li WL (2008) Testing the reliability of genetic methods of species identification via simulation. Syst Biol 57: 216–230. 18398767 10.1080/10635150802032990

[50] Koo I, Zhang X, Kim S (2011) Wavelet- and fourier-transform-based spectrum similarity approaches to compound identification in gas chromatography/mass spectrometry. Anal Chem 83: 5631–5638. 21651237 10.1021/ac200740w PMC3136582

[51] Bruce SJ, Tavazzi I, Parisod V, Rezzi S, Kochhar S, et al (2009) Investigation of human blood plasma sample preparation for performing metabolomics using ultrahigh performance liquid chromatography/mass spectrometry. Anal Chem 81: 3285–3296. 19323527 10.1021/ac8024569

