# Supplementary figures and images for: Forensic DNA Barcoding and Bio-Response Studies of Animal Horn Products Used in Traditional Medicine
Source: PLoS One. 2013 Feb 8;8(2):e55854. doi: 10.1371/journal.pone.0055854 (PMC3568084; doi:10.1371/journal.pone.0055854)

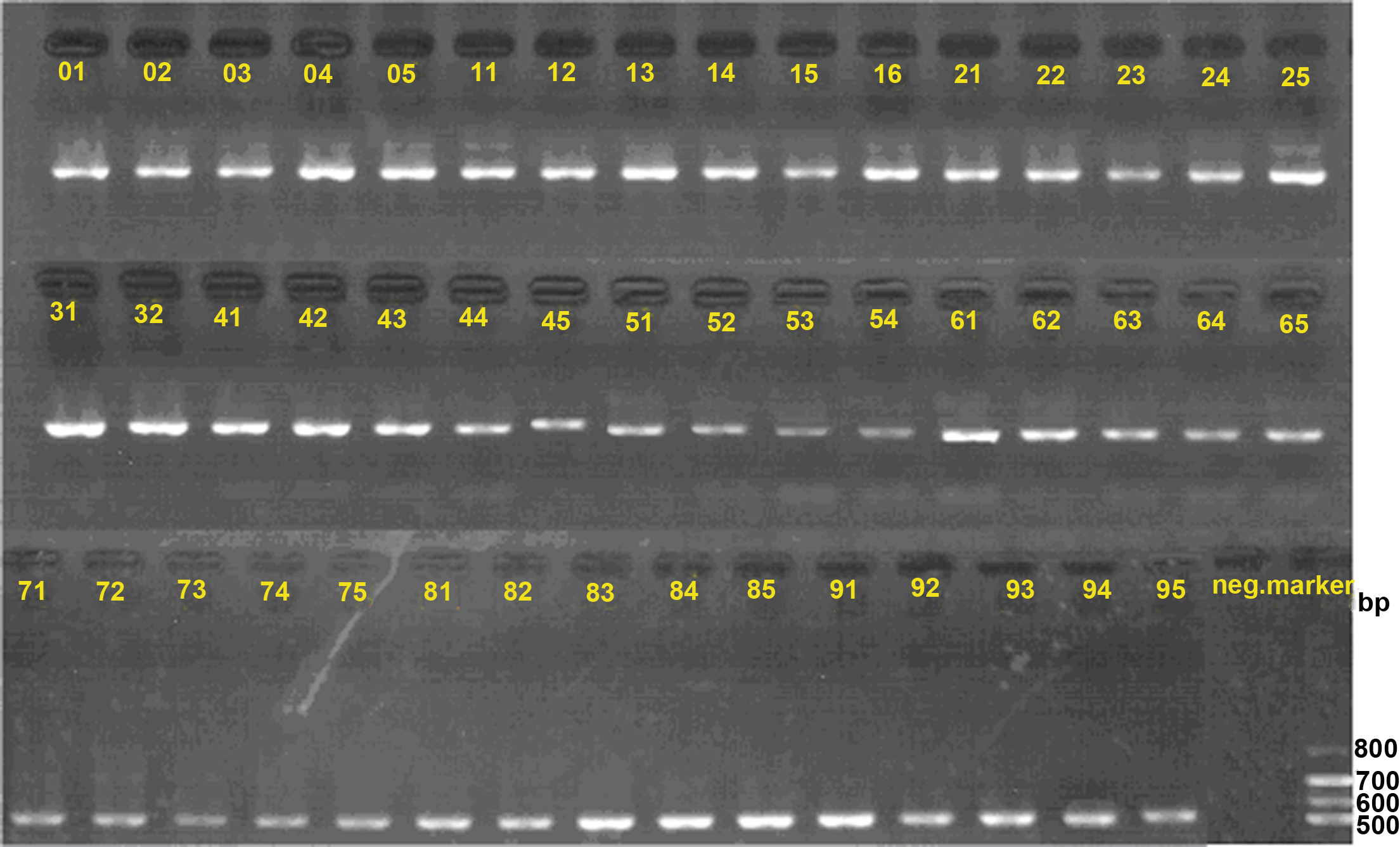

Supplement: Figure S1 — PCR amplication results of COI. 01–05 Red Deer, 11–16 Sika Deer, 21–25 Père David's Deer, 31–32 Saiga Antelope, 41–45 Mongolian Gazelle, 51–54 Domestic Yak, 61–65 Domestic Cattle, 71–75 Asian Water Buffalo, 81–85 Domestic Goat, and 91–95 Domestic Sheep. (TIF) [file pone.0055854.s001.tif]

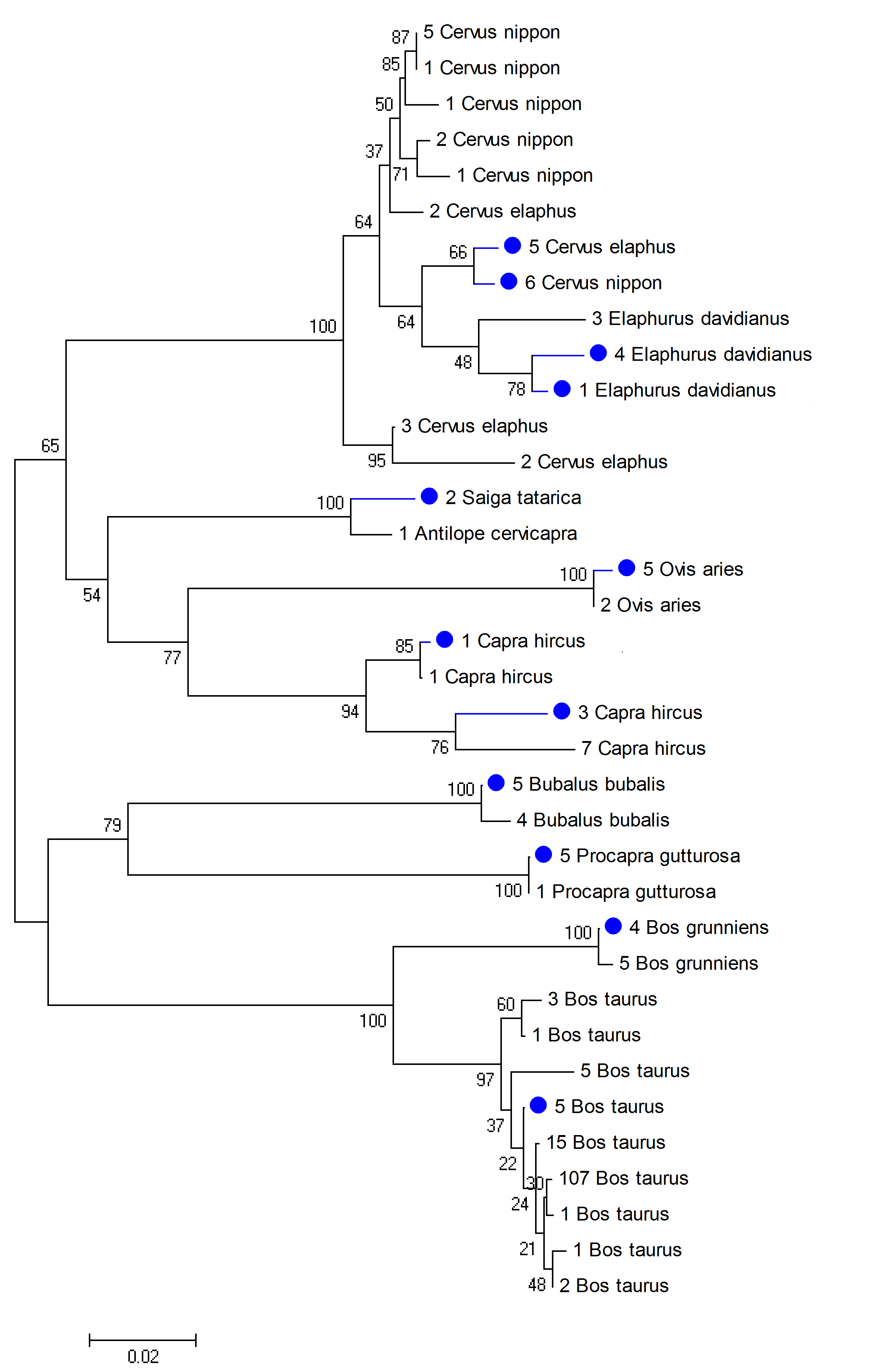

Supplement: Figure S2 — Neighbor-joining tree of 223 COI complete gene sequences available at GenBank. The blue circle represents the experimental individuals; the numbers in front of the taxon names are the species identification numbers. (TIF) [file pone.0055854.s002.tif]

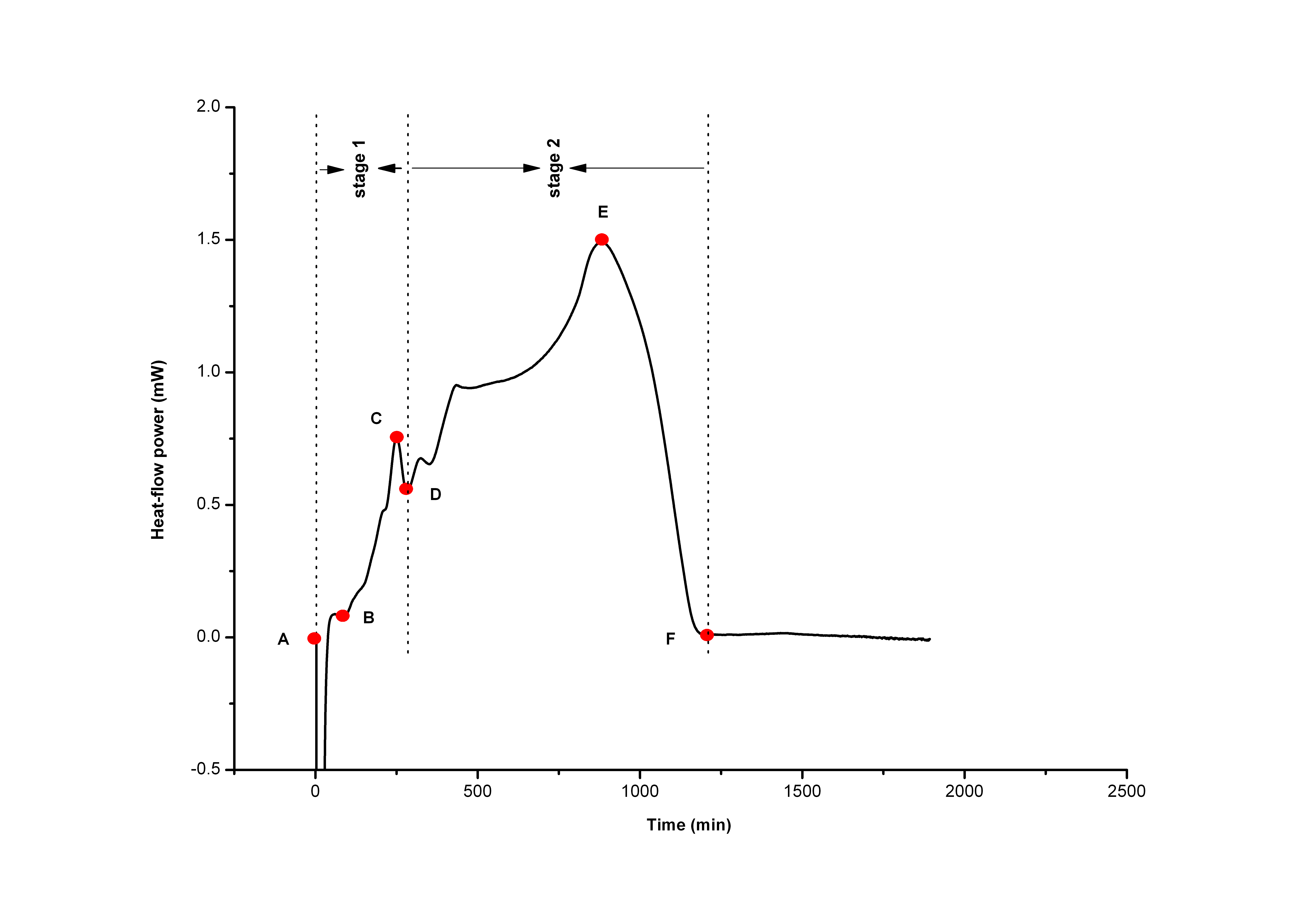

Supplement: Figure S3 — HFP- t curve in the absence of animal horn products. (TIF) [file pone.0055854.s003.tif]

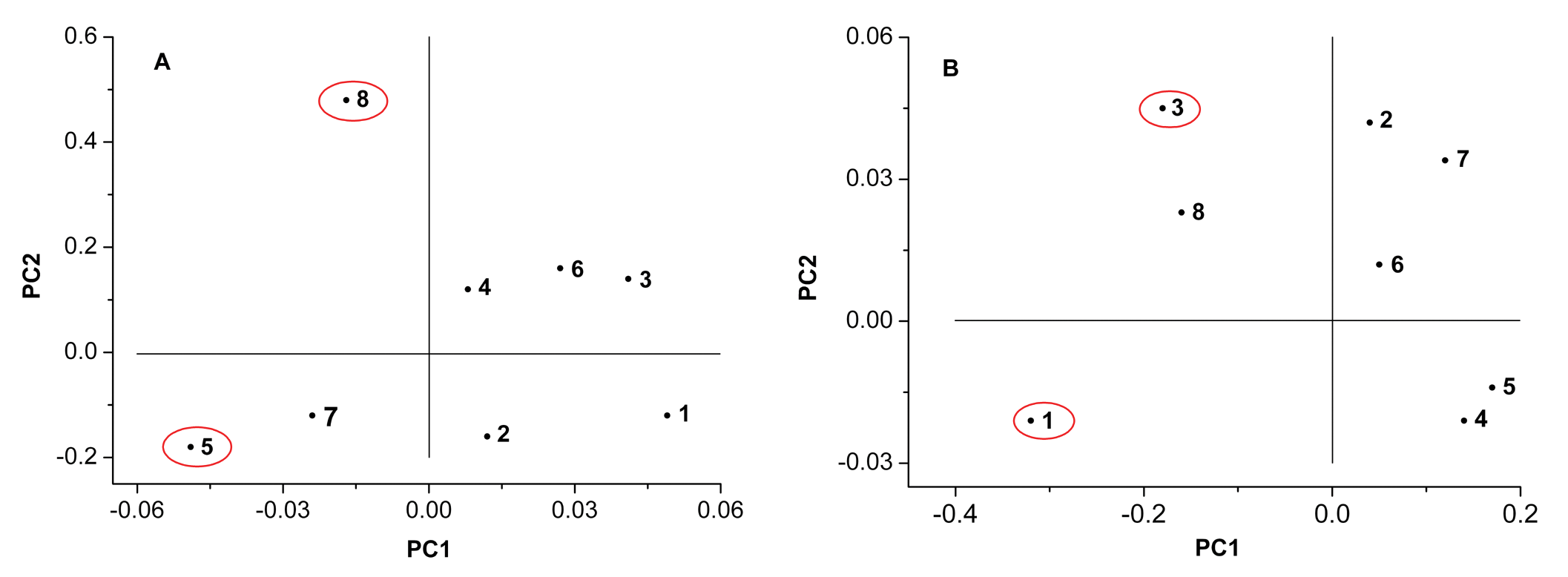

Supplement: Figure S4 — Score plots generated from PCA of the eight quantitative parameters obtained from the HFP- t profiles. (A) Score plots for Red Deer (main parameter P 2 and Q sta, 2). (B) Score plots of Saiga Antelope (main parameter k 1 and T 1). (TIF) [file pone.0055854.s004.tif]

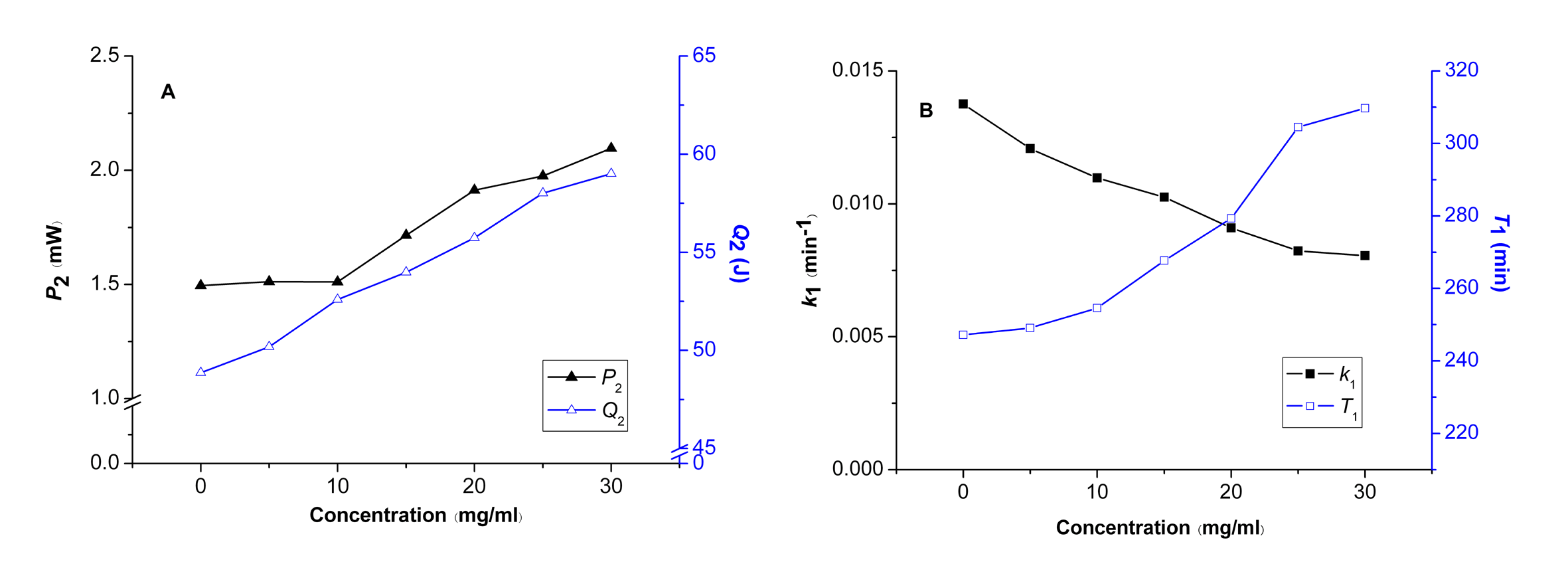

Supplement: Figure S5 — Relationship between the main parameters and the concentration ( c ) of the animal horn samples. (A) relationship between P 2, Q sta, 2 and c for Red Deer. (B) relationship between k 1, T 1 and c for Saiga Antelope. (TIF) [file pone.0055854.s005.tif]
